# Proceedings of Veterinary Medical Societies

**Published:** 1888-04

**Authors:** 


### PROCEEDINGS VETERINARY SOCIETIES.

1. SEMI-ANNUAL MEETING OF THE UNITED STATES VETERINARY MEDICAL ASSOCIATION.—The United States Veterinary Medical Association held its 24th semi-annual meeting at the rooms of the Medico-Chirurgical Faculty of Maryland, in the Athenæum Building, Tuesday, March 20th. The meeting was called to order by the President, Dr. Huidekoper, who was obliged immediately, on account of a severe laryngitis, to request Dr. W. B. E. Miller, to take the chair.

Over thirty members were present, to which number were added several prominent members of the Medical Faculty of Baltimore. Dr. S. K. Johnson, Chairman of the Library Committe, reported their stereotyped motto of "progress." In the absence of Dr. Coates, the Chairman, Dr. J. Faust reported for the Intelligence and Education Committe, a very satisfactory condition of the profession in New York, and the rapid increase of the graduates from the New York Schools.

The Special Committee to secure a uniform Standard in Veterinary Schools, reported that some five schools in the United States and Canada, had returned favorable answers, and that the American Veterinary College of New York had announced a three years course for the coming year.

Dr. Zuill made a short report for the Committee on Contagious Diseases, dwelling especially on the lung-plague and glanders,

Dr. Hoskins, of Pennsylvania, and Dr. Dougherty, of Maryland, were the only State Secretaries present, and they made short reports, which were supplementary to matters already under consideration.

For the Committee on Army Legislation, Dr. Pendry reported that a bill had been introduced in the House of Representatives at Washington, by the Honorable Mr. Steele, of New York, that said bill had passed second reading and had been sent to the Committee on Military affairs; some slight changes had been made from the bill, read and approved at a previous meeting of this association. A resolution was passed endorsing the policy of the Bureau of Animal Industry, and requesting Congress to keep the control of the animal diseases under this Bureau. A resolution was passed thanking the Medico-Chirurgical Faculty, for their courtesy in giving the use of their rooms.

Upon recommendation of the Comitia Minora, the following gentlemen were elected members of the association: James A. Breakel, N. Y.; James McKee, Staten Island; George Bridges, Norwalk; A. C. Young, R. L. Tucker, Mt. Holly, N. Y.; J. D. Fair, Berlin, O.; Philip Neuman, G. C. Faville, Baltimore; E. M. Beckley, Meriden; Alpheus Tuttle, New Briton, Conn.; Chas. Williams, Philadelphia.; W. A. Hitchcock and Harrison Whitney, Malden, Mass.; E. C. Shroeder, Baltimore; D. D. Emerson and E. W. Roche, Boston; E. R. Mercer, Montclair, N. J.; J. Morice, New Orleans; John Wende, Buffalo; A. K. Robertson, N.Y.; John P. Bardan, N. H. Paaren, N.Y., and J.C. Jackson, Yonkers. Also upon recommendation of the Comitia Minora, Dr. D. M. Schaffer, of Indiana, was expelled from the association by a unanimous vote, for unprofessional advertising.

II. Ohio State Veterinary Medical Association, held its fifth annual meeting January 10, 1888, at Akron. President, Dr. J. C. Meyer, Jr., of Cincinnati, called the meeting to order and delivered a brief address, appropriate to the occasion. Twenty-three members present.

The following gentlemen were then elected officers for the ensuing year:

*President*, J. S. Butler, V. S., Piqua.

*First Vice-President*, W. R. Howe, V. S., Dayton.

*Second Vice-President*, W. H. Gribble, D. V. S., Washington C. H.

*Third Vice-President*, J. N. Miller, V. S., Seville.

*Recording Secretary*, W. Shaw, V. S., Dayton.

*Corresponding Secretary*, G. W. Butler, V. S., Circleville.

*Treasurer*, J. V. Newton, V. S., Toledo.

*Board of Censors*, W. F. Derr, V. S., Wooster; J. D. Fair, D.V.S., Berlin, J. C. Meyer, Jr., D. V. S., Cincinnati, and W. R. Howe, V. S., Dayton.

After recess the newly-elected President began his official duties by calling the meeting to order, and in a few appropriate remarks thanked those present for the honor they had conferred upon him.

Notice was given by Dr. J. C. Meyer, Jr., of a previous motion to make some changes in the constitution and by-laws. Drs. Newton, Whitehead, Howe, Meyer, Jr., and J. D. Fair, were appointed as a committee, by the President, to revise and make such changes in the Constitution, By-Laws and Code of Ethics, as they deemed advisable, and submit their report to the next meeting for consideration.

O. J. Carter, V. S., Toledo, T. N. McDermott, V. S., New Philadelphia, and C. Crisman, V. S., Akron, were then proposed as new members. As all were regular graduates, a vote for their admission was immediately taken by ballot and they were unanimously accepted.

Dr. T. S. Butler, of Iowa City, Iowa, tendered his resignation, having permanently removed from the State. The resignation was accepted, and the Secretary instructed to notify Dr. Butler of the same, also to tender him the regret of this Association, for having lost such a valuable member, and to extend to him the best wishes of the Association for his future health and prosperity.

Dr. J. D. Fair, read a complete and exhaustive paper on the "Differential Symptoms of Glanders, Nasal Catarrh, Pus in the Frontal Sinuses, and Decayed Teeth." The paper gave rise to a lengthy and animated discussion, which was chiefly confined to the various forms of Glanders and the nature of the virus, a few of those present advocating that the virus is volatile, while the majority interposed strenuous objections to the acceptance of such a theory.

Dr. W. G. Torrance read an essay on "The Use of Electricity in Veterinary Practice." The paper gave evidence of a great amount of labor in its preparation, and was entitled to the highest commendation.

An instructive paper on "Tetanus," by Dr. R. W. Whitehead, was thoroughly discussed, the majority of those present taking part. One among the many features of the disease alluded to in the discussion was the germ theory. It was also quite evident from he facts adduced that tetanus is much more prevalent in some counties than in others, as some of the members present, through a practice of many years, had only met with a few cases, while others had seen a greater number in a single season.

Dr. J. N. Miller exhibited a large "Pharyngeal Polypus," which he removed from an animal whose misfortune had been the cause of it having fallen into the hands of numerours traders, but after the operation it became a useful and valuable animal.

Dr. W. H. Gribble, in a paper on "Actinomykosis," ably explained the nature and progress of the disease in the lower animals and in man, and the discussion to which the paper gave rise was both interesting and instructive.

Dr. E. R. Barnett read an interesting paper on a case of "Volvulus," which came under his observation.

Dr. J. D. Fair was appointed by the Association to attend the next meeting of the United States Veterinary Medical Association as a visitor for the purpose of inviting said Association to meet the Ohio State Association at its next annual meeting. The meeting then adjourned to meet at Cincinnati in July next.

G. W. BUTLER, V. S.,
*Cor. Secretary.*

III. NEW JERSEY STATE VETERINARY SOCIETY.—Met in convention in the city of Newark, on Thursday, October 27, 1887, with Dr. J. C. Corlies in the chair. Altnough as now incorporated the membership consists exclusively of graduates, yet the attendance was larger than at some of the meetings of the old association, which admitted both graduates and non-graduates to membership, thus showing that the veterinary profession of New Jersey recognizes the imperative necessity of a complete emancipation from quackery and its associations.

The Secretary presented the certified copy of the certificate of incorporation of the NEW JERSEY STATE VETERINARY SOCIETY, which had been received from the Hon. Henry C. Kelsey, Secretary of State at Trenton.

The Secretary had received many letters of encouragement from eminent veterinarians at home and abroad. Letters from the following gentlemen were read amid enthusiasm: Professor Liautard, Dean of the American Veterinary College; Professor McEachran, Principal of the Montreal Veterinary College; Professor Chas. P. Lyman, of the Veterinary Department of Harvard University; Professor James Law, of Cornell University; Professor Huidekoper, of the Veterinary Department of the University of Pennsylvania; Professor James L. Robertson, of the American Veterinary College: Professor A. H. Baker, of the Chicago Veterinary College; Dr. Ezra M. Hunt, Secretary of the New Jersey State Board of Health; Professor Chas. B. Michener, of the American Veterinary College; Dr. George Fleming, of London, England, and others.

Appropriate resolutions were passed in the matter of the death of Dr. Edmund Chambon, of Jersey City, one of the men who rendered material aid in the establishment of the Association on its present basis. Dr. Chambon stood high in his chosen profession, he having graduated with distinction at the Imperial Veterinary School at Alfort, France.

The Board of Censors reported favorably on the application of Dr. W. H. Mook, of Metuchen, and he was unanimously elected to membership.

An able address was delivered by President Corlies on the recent equine epidemic in New Jersey. While he admitted that isolated cases of spinal meningitis occurred in differents parts of the State, yet he considered that it was a misnomer to apply this term to the recent outbreak. He did not think that there was any name for it that was comprehensive, consequently he had coined a name himself, *Carbo-Hæminia.* The doctor claimed that the disease was due to an excess of carbonic acid generated in the system, that the blood was not oxygenized. He had made two post-mortems and found the blood of a venous character, intensely black with antemortem heart clot, together with other marked lesions of this character. There were no traces of lesions of the brain or spinal cord.

Dr. Corlies' remarks led to a prolonged and beneficial discussion on differential diagnosis between spinal meningitis, typhoid influenza, and carbo-hæminia, in which Doctors Nayler, Mook, Sherk, Krowl, Vogt, Mercer, Sattler, DeClyne and Lowe took part. So much interest was taken and clinical experience related that some of those present thought that the time had been better occupied than if they had listened to an elaborate essay.

The subject of Veterinary Legislation again received a due amount of attention, and it was the sense of the meeting that the proposed bill should be modified and drafted so that both classes of practitioners would be allowed to register—the graduates as "Regular Practitioners," and the non-graduates as "Existing Practitioners," but that the time of allowing the latter class to register should be limited to six months after the passage of the act. By this method all those who have assumed the right and are practicing for a livelihood would be allowed to register as "Existing Practitioners," provided they take advantage of the opportunity and do so within six months after the passage of the act. This measure was considered a just one, for while it did not prevent the non-graduate from making a living, yet it made a distinction between the graduate and the non-graduate. If the time for the registering of "Existing Practitioners" was limited to six months, after that time had elapsed, a complete register of such practitioners could be obtained as well as of the "Regular Practitioners." The limiting of the registration of non-graduates to a specified time was regarded as essential if benefit is to be derived from the legislation. While it is desirable to stop the "sailing under false colors," yet there is nothing in the bill to prevent a farmer, or in fact any person, from treating a sick animal so long as he does not represent himself as a veterinary surgeon.

It was decided to hold the next meeting of the Association in Trenton. A banquet was held in the evening.

WM. HERBERT LOWE, D.V.S., *Secretary.*

IV. COLORADO STATE VETERINARY ASSOCIATION.—On Wednesday evening, February 1, a meeting of the graduated veterinary surgeons of the State was held at the office of Dr. Chas. G. Lamb, for the purpose of forming the Colorado State Veterinary Medical Association, at which meeting the following officers were elected:

President, Dr. William McEachran; Vice-President, Dr. A. F. Martins; Secretary and Treasurer, Dr. Charles G. Lamb.

The objects of the Association are mutual benefit and the advancement of veterinary science in this State.

The charter members are Drs. A. F. Martins, William McEachran, C. L. Smith, Sol Bock and Charles G. Lamb.

V. ONTARIO VETERINARY MEDICAL ASSOCIATION.—The annual meeting of this Association was held on the 22d of December, in the Museum of the Ontario Veterinary College. The association is now a large one, the members hailing from all parts of the continent.

The meeting was called to order by the retiring President, Wm. Shaw, V. S., of Dayton, Ohio, whose address, passing in review matters of interest to the veterinary world, which had occurred during his term of office, was full of interest.

After routine proceedings, R. J. Quinn, V. S., of Brampton, Ontario, was called upon, and delivered an address on the castration of Ridglings. Mr. Quinn's experience has been large, and his ideas commended themselves to many of the members, although others as vigorously dissented from parts of the address.

Among other papers of interest, were one by J. F. Smith, V. S., of Brantford, Ontario, and one by G. Bell, V. S., of Lockport, N. Y.

The following officers were elected: Honorary President, Professor Smith; President, T. C. Grenside, V. S., of Guelph, Ontario; 1st Vice-President, R. J. Quinn, V. S., Brampton, Ontario; 2d Vice-President, J. Dunphy, V. S., of Salford, Ontario; Secretary, C. Sweetapple, V. S., of Oshawa, Ontario; Treasurer, W. Cowan, V. S., of Galt, Ontario; Directors, D. M'Intosh, V. S., D. McArthur, V. S., D. Hamilton, V. S., W. J. Wilson, V. S., J. F. Smith, V. S., W. H. Burns, V. S., J. W. Faskin, V. S., and G. Y. Ormsby, V. S.

Auditors, J. O'Niel, V. S., and C. Elliott, V. S.

VI. AMERICAN VETERINARY COLLEGE.—The commencement exercises of this college took place in New York the 1st of March, at Chickering Hall. The President of the Board of Trustees delivered the diplomas to the successful candidates, as their names were called by the Dean of the Faculty, Prof. A. Liautard. Rev.

Dr. Deems, of the Church of Strangers, delivered a most interesting address. The prizes were delivered by Prof. Doremus, as follows: The Trustees, prize to M. W. Tritschler, D. V. S.; the Faculty, or Practical prize, to H. B. Ambler, D. V. S.; the Alumni prize, to J. F. Pease, D.V. S.; the Anatomical prize to T. M. Buckley, D. V. S. Thirty-two graduates received their degree of Doctor of Veterinary Surgery (D. V. S. :

Henry Babcock Ambler, New York.
Harry Macfadden Ball, Ohio.
John Linn Bradley, Pennsylvania.
Thomas Michael Buckley, New York.
Bernard Henry Bueter, Kentucky.
Myron Emerson Chapin, Massachusetts.
Elbert James Decker, New York.
Arthur Leroy Dodge, New Hampshire.
William Albert Engeman, New York.
Otto Faust, New York.
Alexander Fletcher, Ohio.
Frank Branco Ford, West Virginia.
James Walter Harwood, Illinois.
Adolph Carl Hexamer, New York.
Daniel R. Hoffman, Maine.
Frederick Wilcox Hunt, New York.
Charles Clow Jackson, Missouri.
Gilbert Azur Lathrop, Pennsylvania.
Wm. Herbert Lowe, D. V. S., New Jersey.
Harris B. McDowell, Delaware.
Albert Hopper McIntosh, New Jersey.
David William McKillop, Illinois.
James Frederick Pease, Illinois.
Cyrus Polk, Delaware.
George Augustus Smith, New York.
Alfred Isaac Spurr, Massachusetts.
John James Streets, Illincis.
Morris William Tritschler, Pennsylvania.
Robert Addy Van Nest, Minnesota.
Edgar Avery Vreeland, New Jersey.
John Wright Wilkinson, Pennsylvania.
John William Henry Wright, New York.

VII. New York College of Veterinary Surgeons. — The thirty-first annual commencement of the New York College of Veterinary Surgeons and School of Comparative Medicine, was held in the Carnegie Laboratory of Bellevue Hospital Medical College, on Wednesday evening, March 14th, and was attended by a select audience. The excercises were enlivened by excellent music. Dr. Herman M. Biggs, of Bellevue College, delivered the address of the evening.

The President of the College, Dr. Wm. T. White, conferred the degree of Veterinary Surgeon on the following successful students:

M. Kenny, New York.
R. McLean, "
Chas. Schlosser, "
P. Burns, "
Jas. Kelly, "
Geo. H. Roberts, "
B. G. Orlapp, Missouri.
T. S. Culbert, Indiana.
S. S. Mayer, Pennsylvania.
Wm. B. Prathero, "
Henry Henning, "
Aug. Tasme, Georgia.

A majority of junior students, having passed their first examination on Anatomy, Materia Medica, Physiology and Chemistry, were awarded the junior certificate.

M. Kenny was awarded the first prize, a gold medal, for best general examination. Henry Henning received first prize in Anatomy, also a special prize for the best mounted skeleton of a horse. Aug. Tasme was awarded a silver medal for injected specimen. Geo. H. Roberts of the graduating class, delivered the valedictory. The proceedings terminated by a banquet, given by the graduating class, at which the faculty and friends of the college and students were present.

VIII. Ontario Veterinary College.—The Government Board for the examination of veterinary students met in one of the lecture rooms of the Ontario Veterinary College on the 21st of December. A good number of gentlemen presented themselves as aspirants for a veterinary diploma. The large majority of those going up acquitted themselves with credit, showing that they had been thoroughly trained both in the theory and practice of their chosen profession. Twenty-four gentlemen passed, and were granted diplomas. Their names are given alphabetically :

Baxter, G., Michigan.
Broad, W. F., Senya.
Clapp, W. H., Senya.
Cooper, H., Davisville.
Collins, C. O., Obolds, Pa.
Coates, R. C., Bothwell.
Carpenter, W. H., Holly, N. Y.
Cunningham, E. E., La Porte, Ind.
Dean, H., Navistock.
Ewing, W. A., Newmarket.
Evans, W. M., Simcoe.
Huck, W. H, Mildmay.
Kannon, M., Montreal, Que.
Kumpf, W. A., Waterloo.
Kintner, S. P., Wooster, Ohio.
McMurray, O. M., North Baltimore. O.
McLaren, C. L., Highgate.
Oyler, J. H., Harrisburg, Pa.
Pike, F., Toronto.
Shillinglaw, W., Staffo.
Story, R. W., Princeton, Ill.
Taylor, B., Hillsboro, Dak.
Thomson, W., Arillia.
Walker, R. J., Clogher, Ireland.
Bracker, G. E., Primary on Materia Medica.
Widdifield, R. J., Primary on Materia Medica.
Teller, J. H., Primary on Anatomy.

IX. The New York State Academy of Veterinary Science and Comparative Pathology.—Held its annual meeting and dinner at the United States Hotel. At the meeting the following officers were installed : President, Dr. J. M. Heard ; First Vice-President, Dr. Edward S. Breder ; Second Vice-President, Dr. H. E. Earl ; Secretary, Dr. H. D. Gill ; Treasurer, Dr. J. Hamill. The academy at present has thirty-two members. Twenty-five gentlemen sat down to the dinner which followed the meeting. Among them were Drs. J. Ward, of Baltimore, Plageman, Lambert, Robertson, and Prof. Garrish. The following toasts were responded to : "The Academy of Veterinary Science and Comparative Pathology," Dr. James Hamill ; "The Veterinary Profession," Dr. Ward, "Our Visitors," Dr. W. Hassal of Baltimore.

